# Preterm birth is strongly affected by the glucocorticoid dose during pregnancy in women complicated by systemic lupus erythematosus

**DOI:** 10.1186/s13075-021-02699-1

**Published:** 2022-01-03

**Authors:** Hiromi Shimada, Risa Wakiya, Kenji Kanenishi, Nobuyuki Miyatake, Shusaku Nakashima, Mai Mahmoud Fahmy Mansour, Mikiya Kato, Taichi Miyagi, Koichi Sugihara, Yusuke Ushio, Rina Mino, Mao Mizusaki, Tomohiro Kameda, Norimitsu Kadowaki, Hiroaki Dobashi

**Affiliations:** 1 Department of Internal Medicine, Division of Hematology, Rheumatology and Respiratory Medicine, Faculty of Medicine, Kagawa University, 1750-1, Ikenobe, Kida-gun, Miki-cho, Kagawa Japan; 2 Department of Perinatology and Gynecology, Faculty of Medicine, Kagawa University, Kagawa, Japan; 3 Department of Hygiene, Faculty of Medicine, Kagawa University, Kagawa, Japan

**Keywords:** Glucocorticoid, Systemic lupus erythematosus, Adverse pregnancy outcome, Preterm birth

## Abstract

**Background:**

This study aimed to investigate the effect of glucocorticoid doses on adverse pregnancy outcomes (APOs) in women complicated by systemic lupus erythematosus (SLE).

**Methods:**

We investigated 74 pregnancies complicated by SLE or SLE-dominant mixed connective tissue disease. The pregnancies were managed from conception to delivery in our institution. We retrospectively evaluated whether the mean glucocorticoid dose during pregnancy is associated with APOs, including preterm birth (PB), low birth weight (LBW), and light-for-date (LFD). We also calculated the cut-off dose of glucocorticoid that affected APOs.

**Results:**

All APOs occurred in 35 (50.7%) patients, with 14 cases of PB, 23 cases of LBW, and 10 cases of LFD. Patients with all APOs or PB had a higher dose of glucocorticoid during pregnancy than patients without all APOs or with full-term birth (*P* = 0.03, *P* <  0.01, respectively). Logistic regression analysis for all APOs and PB showed that the cut-off values of the mean glucocorticoid dose were 6.5 and 10.0 mg/day, respectively. Patients who delivered LBW or LFD newborns showed no significant difference in the glucocorticoid dose used during pregnancy than patients without LBW or LFD newborns. Patients who delivered LBW newborns were more likely to have used glucocorticoids during pregnancy (*P* <  0.01).

**Conclusions:**

In pregnancies complicated by SLE, a relatively lower dose of glucocorticoid than previously reported is significantly related to APOs, especially PB. Therefore, the disease activity of patients with SLE should be managed with the appropriate lower dose of glucocorticoid during pregnancy.

**Supplementary Information:**

The online version contains supplementary material available at 10.1186/s13075-021-02699-1.

## Background

Systemic lupus erythematosus (SLE) occurs in women of child-bearing age. Recently, patients with SLE have been able to be diagnosed earlier according to the diagnosis or classification criteria of each disease and achieve long-term remission by treatment agents, including glucocorticoids and several immunosuppressants. Therefore, women with SLE who used to avoid pregnancy because of their disease, can currently conceive and become mothers. However, women with SLE have more difficulty in achieving a successful pregnancy than healthy women. Women with SLE have a higher risk of adverse pregnancy outcomes (APOs), including preterm birth (PB), light-for-date (LFD), premature rupture of the membranes (PROM), and preeclampsia [1–6]. These APOs are related to uncontrolled high disease activity [7–10]. Therefore, SLE disease activity needs to be strictly controlled with glucocorticoids and immunosuppressants, which are tolerable in pregnancy.

Glucocorticoids are the most commonly used agents for maintaining and managing high aggressive disease activity during pregnancy. The updated British Society for Rheumatology guideline, the European League Against Rheumatism recommendation, and the 2020 American College of Rheumatology (ACR) guideline showed that prednisolone was compatible with each trimester of pregnancy [11–13]. After completion of placental development, maternal glucocorticoids are metabolized by 11-beta-hydroxylase in the placenta, and < 10% of the dose of prednisone or prednisolone crosses the placenta. Several studies have shown that glucocorticoids are not associated with major abnormalities, especially in oral cleft [14, 15]. However, a prolonged high dose of glucocorticoid use during pregnancy increases the risk of APOs. Reinisch et al. showed that intrauterine growth restriction was associated with glucocorticoids, and it was independent of maternal disease activity [16]. Many studies have also shown that steroid use during pregnancy is a risk factor for PB and low birth weight (LBW) [15, 17–19]. Charkravarty et al. found that preterm delivery was also associated with glucocorticoid use in pregnancies complicated by SLE [20]. In addition, high doses of glucocorticoids increase the risk of serious infection during pregnancy [21].

Several reports have also described that using < 20 mg/day of prednisolone during pregnancy and sometimes higher doses of prednisolone are appropriate for aggressive disease activity [22–24]. Palmsten et al. showed that high doses of prednisolone (> 20 mg/day) early and late in pregnancy were associated with PB [25]. Additionally, the ACR guideline showed that glucocorticoids should be tapered to < 20 mg/day by adding pregnancy-compatible immunosuppressants [11]. However, whether a glucocorticoid dose of < 20 mg/day is appropriate for mothers with SLE and infants has not been clarified.

Therefore, this study aimed to investigate the effect of glucocorticoid doses on adverse pregnancy outcomes (APOs), including PB, LBW and LFD, in women complicated by SLE. We also evaluated which glucocorticoid dose can cause these APOs.

## Methods

### Patients and data collection

Our study used data from patients with SLE and those with SLE-dominant mixed connective tissue disease (MCTD) who were treated in Kagawa University Hospital from March 2006 to April 2021. These patients were diagnosed with the 1997 ACR revised criteria for SLE [26] or the 2019 Japanese Ministry of Health, Labour and Welfare diagnostic criteria of MCTD [27]. MCTD was diagnosed with any two characteristics of SLE, myositis, and systemic sclerosis. SLE-dominant MCTD was defined as patients with MCTD who mainly had symptoms of SLE and immunological abnormalities, such as erythema, low complement levels, or a high titer of anti-double stranded antibody. The patients were treated at Kagawa University Hospital from preconception counseling to pregnancy and delivery. We investigated these patients’ clinical background (age at conception disease duration, which was defined as the period from disease onset to conception, and parity), autoantibody profiles (including anti-SS-A/B antibodies and antiphospholipid antibodies), SLE disease activity scores and parameters, and the status of treatment agents (immunosuppressants and glucocorticoid used). The disease activity score was calculated using the SLE Disease Activity Index (SLEDAI) [28, 29] and the lupus low disease activity state (LLDAS) [30]. The parameters of the SLE disease activity score included complement levels of C3, C4, and CH50, and the titer of anti-dsDNA antibody. All laboratory tests were performed using standard methods.

We examined glucocorticoid use at conception or during pregnancy, and the mean dose of glucocorticoid, which was defined as the average glucocorticoid dose from conception to delivery. Additionally, we investigated whether the dose of glucocorticoid needed to be increased during pregnancy.

We evaluated the association between glucocorticoid use and APOs, which included PB, LBW, and LFD. PB was defined as delivery before 37 gestational weeks. LBW was defined as newborns who were born weighing < 2500 g. LFD was defined as newborns whose birth weight was lower than the 10th percentile. PROM was defined as rupture of the membranes (amniotic sac) before labor began, and preterm PROM occurred before 37 gestational weeks.

All data were retrospectively collected from medical records at Kagawa University Hospital. This was a retrospective, observational study. Therefore, we did not require ethical approval. We did not obtain informed consent from each patient, however, we disclosed the information of our research.

### Statistical analysis

Values are presented as mean ± standard deviation or number (%). We performed univariate analysis to determine the associations between APOs and glucocorticoid use, mean glucocorticoid dose, and increasing the dose of glucocorticoid during pregnancy. A logistic regression analysis of the associations between the glucocorticoid dose and APOs, PB, LBW, and LFD was performed. The receiver operating characteristic (ROC) curve was used to determine the cut-off value for the mean glucocorticoid dose that affected APOs. Descriptive statistics were compared using the Wilcoxon rank sum test for continuous variables and Fisher’s exact test for categorical variables. A two-sided *P* value of < 0.05 was considered significant. We evaluated parameters with significant differences (*P* <  0.05) as risk factors. All analyses were conducted using JMP for Mac, Version 13.0.0 (SAS Institute, Japan).

## Results

### Patients’ characteristics and treatments

Our study used data from 74 pregnancies in 52 patients with SLE and those with SLE-dominant MCTD. The patients’ characteristics, disease activities, treatment agents, and pregnancy outcomes are shown in Table 1. The mean age at delivery was 31.8 ± 4.4 years and the mean disease duration was 10.0 ± 5.1 years. Forty-two (56.8%) patients were positive for anti-SS-A antibody and 29 (39.2%) were positive for antiphospholipid antibodies. With regard to disease activity parameters, the mean SLEDAI score in the first and third trimesters was 1.8 ± 2.1 and 1.2 ± 1.9, respectively. Forty (59.7%) achieved an LLDAS and 50 (74.6%) achieved an LLDAS without a glucocorticoid dose at conception. Complement levels were normal (C3: 89.8 ± 21.4, C4: 18.2 ± 6.7, CH50: 41.0 ± 9.6), and the titer of anti-dsDNA antibody was almost normal (10.8 ± 32.6). Regarding treatment agents used at conception and during pregnancy, immunosuppressants were administered in 16 (21.6%) patients at conception. Glucocorticoids were administered in 57 (77.0%) patients at conception and in 61 (82.4%) patients during pregnancy, and all of them took prednisone. The mean dose of glucocorticoid at conception was 6.5 ± 3.2 mg/day, and that during pregnancy was 8.4 ± 4.8 mg/day. Fifteen (20.3%) patients needed to increase their dose of glucocorticoid during pregnancy because the SLE disease activity was elevated.

**Table 1:** Patients’ characteristics, disease activities, treatment agents, and pregnancy outcomes

	(*n* = 74)	
**Patients’ characteristics**		
**Mean age at delivery, years**	**31.8 ± 4.4**	
**Mean disease duration, years**	**10.0 ± 5.1**	
**Parity, n (%)**	**40 (54.1)**	
**Autoantibody positivity**		
Anti-SS-A antibody, n (%)	42 (56.8)	
Antiphospholipid antibodies, n (%)	29 (39.2)	
LAC/anti-cardiolipin/anti-CLβ2GP1	23 (31.1)/10 (13.5)/3 (4.1)	
Number of positive antibodies (single/double/triple)	22 (29.7)/6 (8.1)/1 (1.4)	
**Disease activity parameters**	**First trimester (at conception)**	**Third trimester**
Mean SLEDAI score	1.8 ± 2.1	1.2 ± 1.9
Achievement of LLDAS, n (%)	40 (59.7)	35 (58.3)
Achievement of LLDAS without a glucocorticoid dose, n (%)	50 (74.6)	50 (83.3)
C3, mg/dl	89.8 ± 21.4	101.9 ± 23.1
C4, mg/dl	18.2 ± 6.7	17.9 ± 7.2
CH50, IU/ml	41.0 ± 9.6	42.7 ± 10.1
Titer of anti-dsDNA antibody, IU/ml	10.8 ± 32.6	5.8 ± 12.3
**Status of treatment agents**		
**Immunosuppressant use at conception, n (%)**	**16 (21.6)**	
**Hydroxychloroquine use during pregnancy, n (%)**	**10 (13.5)**	
**Glucocorticoid use**		
Use at conception, n (%)	57 (77.0)	
Mean dose at conception, mg/day	6.5 ± 3.2	
Use during pregnancy, n (%)	61 (82.4)	
Mean dose during pregnancy, mg/day	8.4 ± 4.8	
Increasing doses used, n (%)	15 (20.3)	
**Pregnancy outcomes**	(*n* = 74)	
**Neonatal loss, n (%)**	**15 (20.3)**	
Spontaneous abortion, n (%)	8 (10.8)	
Stillbirth, n (%)	2 (2.7)	
Induced abortion, n (%)	5 (6.8)	
**Live birth, n (%)**	**59 (79.7)**	
Cesarean section, n (%)	16 (27.1)	
Gestational age at delivery, weeks	37.5 ± 3.0	
Birth weight of newborns, g	2643.3 ± 665.2	
**APOs, n (%)**	**35 (50.7)**	
Spontaneous abortion, n (%)	8 (10.8)	
Stillbirth, n (%)	2 (2.7)	
PB, n (%)	14 (23.7)	
LBW newborns, n (%)	23 (39.0)	
LFD newborns, n (%)	10 (16.9)	
Preterm PROM, n (%)	7 (11.9)	
Preeclampsia, n (%)	5 (8.6)	
NICU administration, n (%)	17 (28.8)	

Values are shown as mean ± standard deviation or n (%). *LAC* lupus anti-coagulant, *CLβ2GP1* cardiolipin-beta 2 glycoprotein 1, *SLEDAI* Systemic Lupus Erythematosus Disease Activity Index, *LLDAS* lupus low disease activity state, *APOs* adverse pregnancy outcomes, *PB* preterm birth, *LBW* low birth weight, *LFD* light-for-date, *PROM* premature rupture of the membranes, *NICU* neonatal intensive care unit

### Pregnancy outcomes

Table 1 also shows the pregnancy outcomes in all patients. Fifteen (20.3%) patients experienced loss of the newborn because of eight (10.8%) cases of spontaneous abortion, two (2.7%) cases of stillbirth, and five (6.8%) cases of induced abortion. In 59 (79.7%) patients with a live birth, the mean gestational age at delivery was 37.5 ± 3.0 weeks. The mean birth weight of newborns was 2643.3 ± 665.2 g. APOs occurred in 35 (50.7%) women, and these included spontaneous abortion, stillbirth, 14 (23.7%) cases of PB, 23 (39.0%) cases of LWB, 10 (16.9%) cases of LFD, 7 (11.9%) cases of preterm PROM, and 5 (8.6%) cases of preeclampsia.

### Analysis of risk factors for APOs

**Fig. 1:**
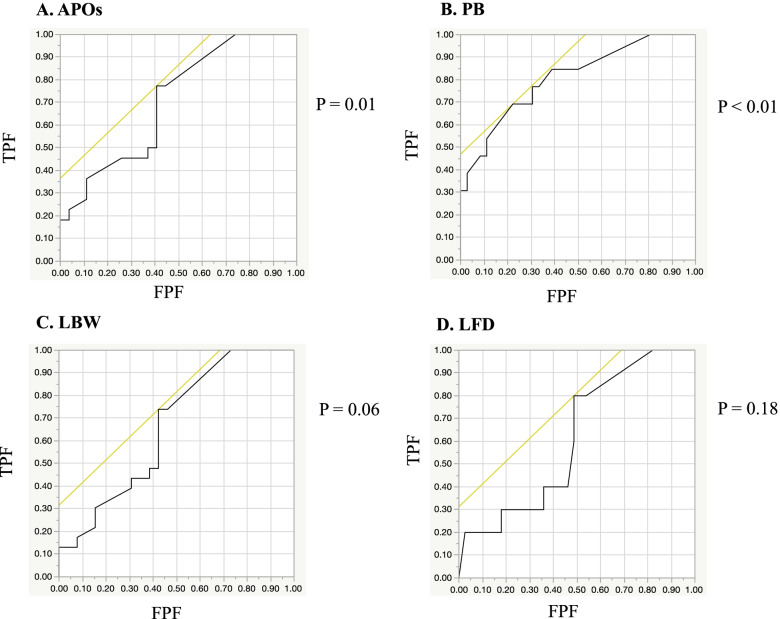
ROC curves based on logistic regression analysis of cut-off values for the mean glucocorticoid dose. **A**. ROC curve for APOs (*P* = 0.01). The AUC was 0.674 and the cut-off value for the mean prednisone dose was 6.5 mg/day. **B**. ROC curve for PB (*P* < 0.01). The AUC was 0.808 and the cut-off value for the mean prednisone dose was 10.0 mg/day. **C**. ROC curve for LBW (*P* = 0.06). The AUC was 0.662 and the cut-off value for the mean prednisone dose was 6.5 mg/day. **D**. ROC curve for LFD (*P* = 0.18). The AUC was 0.615 and the cut-off value for the mean prednisone dose was 6.7 mg/day. ROC: receiver operating characteristic; AUC: area under the curve; TPF: true positive fraction; FPF: false positive fraction; APOs: adverse pregnancy outcomes; PB: preterm birth; LBW: low birth weight; LFD: light-for-date

We investigated the associations between glucocorticoid use and all APOs, PB, LBW, and LFD in all patients (Table 2). All APOs were evaluated in 69 patients after excluding 5 cases of induced abortion. Additionally, PB, LBW, and LFD were examined in 59 patients after excluding 8 cases of spontaneous abortion and 2 cases of stillbirth. The mean glucocorticoid dose during pregnancy was significantly higher in patients who had all APOs or PB than in patients without all APOs or with full-term birth (*P* = 0.03 and *P* <  0.01, respectively). The rate of increasing the glucocorticoid dose was also significantly higher in patients with all APOs or PB than in patients without all APOs or with full-term birth (*P* = 0.01 and P <  0.01, respectively). We performed logistic regression analysis of the mean glucocorticoid dose for APOs and PB (Fig. 1). The ROC curve showed that the area under the curve (AUC) was 0.674 and 0.808 for all APOs and PB, respectively. The cut-off value of the mean glucocorticoid dose was 6.5 and 10.0 mg/day, respectively (*P* = 0.01 and *P* <  0.01). In patients who had a newborn with a LBW, the rates of glucocorticoid use and increasing doses of glucocorticoids during pregnancy were significantly higher than those in patients with newborns without LBW (both P <  0.01). However, there was no significant difference in the mean glucocorticoid dose between these two groups (*P* > 0.05). There were no significant differences in the rate of glucocorticoid use, mean glucocorticoid dose, or the rate of increasing dose of glucocorticoid during pregnancy between patients with newborns who were LFD and those with newborns who were not LFD (*P* = 0.19, *P* = 0.26, and *P* = 1.00, respectively). In logistic regression analysis for LBW and LFD, the ROC curve showed that the AUC was 0.662 and 0.615, respectively, but they were not significant (*P* = 0.06 and *P* = 0.18, respectively, Fig. 1). Additionally, the cut-off value of the mean glucocorticoid dose was 6.5 and 6.7 mg/day, respectively. In patients who had preterm PROM or preeclampsia, there was no significant difference in the rate of glucocorticoid use or the mean glucocorticoid dose during pregnancy compared with those without preterm PROM or preeclampsia (Additional file 1).

**Table 2:** Associations of glucocorticoid use with APOs, PB, LBW, and LFD

	APOs			PB			LBW			LFD		
	APOs (+) (*n* = 35)	APOs (−) (*n* = 34)	*P* value	PB (+) (*n* = 14)	PB (−) (*n* = 45)	*P* value	LBW (+) (*n* = 23)	LBW (−) (*n* = 36)	*P* value	LFD (+) (*n* = 10)	LFD (−) (*n* = 49)	*P* value
Glucocorticoid use, n (%)##	31 (88.6)	25 (73.5)	0.13	13 (92.9)	36 (80.0)	0.42	23 (100.0)	26 (72.2)	< 0.01*	10 (100.0)	39 (79.6)	0.19
Mean glucocorticoid dose, mg/day#	9.6 ± 5.0	6.6 ± 3.7	0.03*	12.3 ± 5.5	6.7 ± 3.3	< 0.01*	9.5 ± 4.9	7.1 ± 4.2	0.05	10.0 ± 5.7	7.8 ± 4.3	0.26
Increase in the glucocorticoid dose, n (%)##	11 (31.4)	2 (5.9)	0.01*	9 (64.3)	3 (6.7)	< 0.01*	9 (39.1)	3 (8.3)	< 0.01*	2 (20.0)	10 (20.4)	1.00

Values are shown as mean ± standard deviation or n (%). #Wilcoxon rank sum test; ##Fisher’s exact test; *P < 0.05. *APOs* adverse pregnancy outcomes, *PB* preterm birth, *LBW* low birth weight, *LFD* light-for-date

We also investigated the associations between disease activity parameters and all APOs, PB, LBW, and LFD in all patients (Table 3). All APOs were significantly associated with achievement of an LLDAS without a glucocorticoid dose at the third trimester (*P* = 0.01), the SLEDAI score at the first and third trimesters (P = 0.01 and *P* = 0.02, respectively), and C3 levels at the first trimester (*P* = 0.01). PB was significantly associated with achievement of an LLDAS at the third trimester (*P* = 0.01), achievement of an LLDAS without a glucocorticoid dose at the first and third trimesters (both of *P* <  0.01), the SLEDAI score at the first trimester (P <  0.01), C3 levels at the first and third trimesters (*P* = 0.02 and P <  0.01, respectively), CH50 levels at the third trimester (P = 0.02), and the titer of anti-dsDNA antibody at the first trimester (*P* = 0.01). LBW was significantly associated with achievement of an LLDAS without glucocorticoid at the third trimester (*P* <  0.01), the SLEDAI score at the first and third trimesters (*P* = 0.03 and *P* = 0.02), and C3 and CH50 concentrations at the first trimester (both *P* = 0.01). However, there was no significant association between LFD and any disease activity parameters.

**Table 3:** Association between disease activity parameters and APOs, PB, LBW, and LFD

		APOs			PB			LBW			LFD		
		APOs (+) (*n* = 35)	APOs (−) (*n* = 34)	*P* value	PB (+) (*n* = 14)	PB (−) (*n* = 45)	*P* value	LBW (+) (*n* = 23)	LBW (−) (*n* = 36)	*P* value	LFD (+) (*n* = 10)	LFD (−) (*n* = 49)	*P* value
Achievement of LLDAS, n (%)##	1st trimester	17 (56.7)	21 (65.6)	0.60	5 (41.7)	29 (70.7)	0.09	12 (63.2)	22 (64.7)	1.00	6 (60.0)	28 (63.6)	1.00
	3rd trimester	12 (46.2)	23 (67.7)	0.12	4 (28.6)	31 (68.9)	0.01*	11 (47.8)	24 (66.7)	0.18	5 (50.0)	30 (61.2)	0.73
Achievement of LLDAS without a glucocorticoid dose, n (%)##	1st trimester	20 (66.7)	27 (84.4)	0.14	5 (41.7)	36 (87.8)	< 0.01*	13 (68.4)	28 (82.4)	0.31	7 (70.0)	34 (77.3)	1.00
	3rd trimester	18 (69.2)	32 (94.1)	0.01*	7 (50.0)	42 (93.3)	< 0.01*	15 (65.2)	34 (94.4)	< 0.01*	8 (80.0)	41 (83.7)	0.67
SLEDAI score#	1st trimester	2.5 ± 2.4	1.0 ± 1.4	0.01*	3.5 ± 2.9	1.1 ± 1.3	< 0.01*	2.3 ± 2.1	1.3 ± 2.0	0.03*	1.7 ± 1.4	1.7 ± 2.2	0.57
	3rd trimester	1.9 ± 2.4	0.7 ± 1.2	0.02*	2.4 ± 3.0	0.8 ± 1.2	0.05	2.0 ± 2.5	0.7 ± 1.2	0.02*	1.8 ± 2.0	1.1 ± 1.9	0.16
C3, mg/dl#	1st trimester	82.4 ± 18.9	99.2 ± 21.6	0.01*	77.3 ± 19.0	95.5 ± 20.9	0.02*	80.5 ± 16.3	97.8 ± 22.3	0.01*	85.3 ± 16.7	92.2 ± 22.7	0.52
	3rd trimester	94.2 ± 25.2	107.7 ± 20.0	0.09	85.5 ± 23.4	108.2 ± 20.1	< 0.01*	93.7 ± 24.9	108.3 ± 20.0	0.07	104.3 ± 31.5	102.2 ± 21.7	0.47
C4, mg/dl#	1st trimester	17.1 ± 6.7	19.6 ± 6.8	0.24	16.1 ± 9.0	19.5 ± 6.2	0.14	17.6 ± 6.3	19.4 ± 7.4	0.47	20.2 ± 7.0	18.3 ± 7.0	0.36
	3rd trimester	16.7 ± 8.4	18.9 ± 6.0	0.19	15.1 ± 6.8	19.1 ± 7.0	0.11	16.9 ± 8.7	18.9 ± 5.9	0.21	20.6 ± 11.2	17.7 ± 6.3	0.51
CH50, IU/ml#	1st trimester	39.1 ± 10.6	43.0 ± 8.4	0.11	37.3 ± 10.6	41.5 ± 8.4	0.13	36.5 ± 6.6	42.7 ± 9.5	0.01*	36.9 ± 9.3	41.1 ± 8.9	0.17
	3rd trimester	40.2 ± 11.5	44.6 ± 8.7	0.18	36.5 ± 10.6	45.0 ± 9.0	0.02*	40.2 ± 11.6	44.8 ± 8.6	0.14	43.8 ± 13.9	42.8 ± 9.4	0.78
Titer of anti-dsDNA antibody, IU/ml#	1st trimester	15.6 ± 44.1	7.0 ± 12.6	0.39	32.5 ± 68.5	5.7 ± 11.2	0.01*	20.7 ± 55.8	7.0 ± 12.3	0.40	30.0 ± 80.0	8.5 ± 14.5	0.94
	3rd trimester	8.7 ± 17.0	3.4 ± 6.1	0.38	14.4 ± 21.4	3.1 ± 5.7	0.05	9.1 ± 17.7	3.6 ± 6.1	0.52	10.3 ± 19.6	5.1 ± 10.9	0.45

Values are presented as mean ± standard deviation or number (%). #Wilcoxon rank sum test; ##Fisher’s exact test; *P < 0.05. *LLDAS* low lupus disease activity status, *SLEDAI* Systemic Lupus Erythematosus Disease Activity Index, *APOs* adverse pregnancy outcomes, *PB* preterm birth, *LBW* low birth weight, *LFD* light-for-date

## Discussion

In this retrospective study, we showed that the mean glucocorticoid dose used during pregnancy was significantly related to APOs, especially in patients who had PB, in pregnancies complicated by SLE and in those with SLE-dominant MCTD. Additionally, women who delivered LBW newborns had a higher rate of glucocorticoid use during pregnancy than those without LBW. We found that the cut-off doses of glucocorticoid that affected all APOs and PB were 6.5 and 10.0 mg/day, respectively. Additionally, a relatively lower dose of glucocorticoid used during pregnancy than that previously reported [22–25] could cause APOs, especially PB. Furthermore, some of the disease activity parameters were associated with APOs, PB, and LBW, but not with LFD.

Previous studies have shown a significant association between connective tissue disease (CTD) and APOs, including PB, LBW, PROM, and preeclampsia [1–4]. In particular, patients with SLE have an increased risk of APOs, which are associated with a high disease activity and disease flare-ups during pregnancy [5–10]. Therefore, the disease activity of SLE needs to be maintained during pregnancy with therapeutic agents that do not affect fetal development. Glucocorticoids are most frequently used for maintenance and flare-up of CTD during pregnancy. In our study, glucocorticoids were administered in approximately 80% of women at conception and during pregnancy. Some patients needed to increase the dose of glucocorticoid and continue a high dose during pregnancy because of their elevated disease activity.

However, prolonged glucocorticoid use during pregnancy is also a risk factor for APOs. Reinisch et al. showed that oral glucocorticoids (especially prednisone) were associated with intrauterine growth restriction in humans and mice, and this was independent of maternal disease [16]. Furthermore, many studies have also shown that steroid use during pregnancy is a risk factor for PB and low birth weight (LBW) [15, 17–19]. Similar to these previous studies, our study showed that women who delivered LBW newborns were more likely to have used glucocorticoids during pregnancy. Additionally, we showed that the mean glucocorticoid dose used during pregnancy was significantly associated with all APOs and PB. However, these APOs were strongly associated with women who had increased doses of glucocorticoids because of an elevated disease activity. We found that some disease activity parameters were significantly related to all APOs, PB, and LBW, but not to LFD. Therefore, APOs are associated not only with glucocorticoid use and the mean glucocorticoid dose, but also with SLE disease activity. Determining whether glucocorticoids or disease activity is the most strongly associated with these APOs is difficult because of the small number of patients and outcomes in our study.

The glucocorticoid dose that can be tolerated in mothers with CTD and their fetus during pregnancy is unclear. Some reports have suggested that the prednisone dose should be < 20 mg/day [22–25]. The 2020 ACR guideline for the management of reproductive health also recommends tapering the prednisone dose to < 20 mg/day by adding pregnancy-compatible immunosuppressants [11]. However, whether < 20 mg/day of prednisone affects APOs is unclear. We found that the cut-off doses of glucocorticoid that affected all APOs and PB were 6.5 and 10.0 mg/day, respectively. The dose that could cause all APOs and PB in our study was relatively lower than that previously reported [22–25]. Therefore, an even lower dose of glucocorticoid < 20 mg/day could be a risk factor for APOs and PB.

Recently, some immunosuppressants have been recognized as safe and compatible during pregnancy. The ACR, European League Against Rheumatism, and British Society for Rheumatology guidelines show the availability of each drug in the period of preconception, during pregnancy, and at breastfeeding [11–13]. These drugs might be helpful in reducing the risk of APOs.

Our study has several limitations. First, our sample comprised a small number of patients, and there was also a small number of outcome events, which might have resulted in a low statistical power. Second, we could not fully exclude the heterogeneity of MCTD. In our study, patients with SLE-dominant MCTD who mainly had SLE symptoms and immunological abnormalities were enrolled, but these patients had some features of myositis or sclerosis. Third, this study was conducted in only one expert institution. Therefore, we might have collected data on patients with relatively high levels of disease activity and risk.

## Conclusions

Continuing glucocorticoid use and the glucocorticoid dose used during pregnancy are significantly associated with APOs, including PB and LBW, in patients with SLE. Additionally, the cut-off doses of glucocorticoid that can cause all APOs and PB are 6.5 and 10.0 mg/day, respectively. Additionally, a relatively lower dose of glucocorticoids used during pregnancy than previously reported is strongly associated with all APOs and PB. Rheumatologists should pay attention to the risk of glucocorticoids in patients with SLE and manage their disease activity with an appropriate dose of glucocorticoid during pregnancy.

## Supplementary Information

**Additional file 1.** Associations of glucocorticoid use with PROM and preeclampsia.

## Data Availability

The data that support the findings in this study will be shared on reasonable request to the corresponding author.

## Abbreviations

APOs: Adverse pregnancy outcomes
ACR: American College of Rheumatology
AUC: Area under the curve
CLβ2GP1: Cardiolipin-beta 2 glycoprotein 1
CTD: Connective tissue disease
FPF: False positive fraction
LAC: Lupus anti-coagulant
LBW: Low birth weight
LFD: Light-for-date
LLDAS: Lupus low disease activity score
MCTD: Mixed connective tissue disease
NICU: Neonatal intensive care unit
PB: Preterm birth
PROM: Premature rupture of the membranes
ROC: Receiver operating characteristic
SLE: Systemic lupus erythematosus
SLEDAI: Systemic lupus erythematosus disease activity index
TPF: True positive fraction
