# Supplementary material for: Preterm birth is strongly affected by the glucocorticoid dose during pregnancy in women complicated by systemic lupus erythematosus
Source: Arthritis Res Ther. 2022 Jan 3;24:10. doi: 10.1186/s13075-021-02699-1 (PMC8722014; doi:10.1186/s13075-021-02699-1)
Supplement: Supplementary file 1 — Additional file 1. Associations of glucocorticoid use with PROM and preeclampsia. [file 13075_2021_2699_MOESM1_ESM.pptx]

## Slide 1
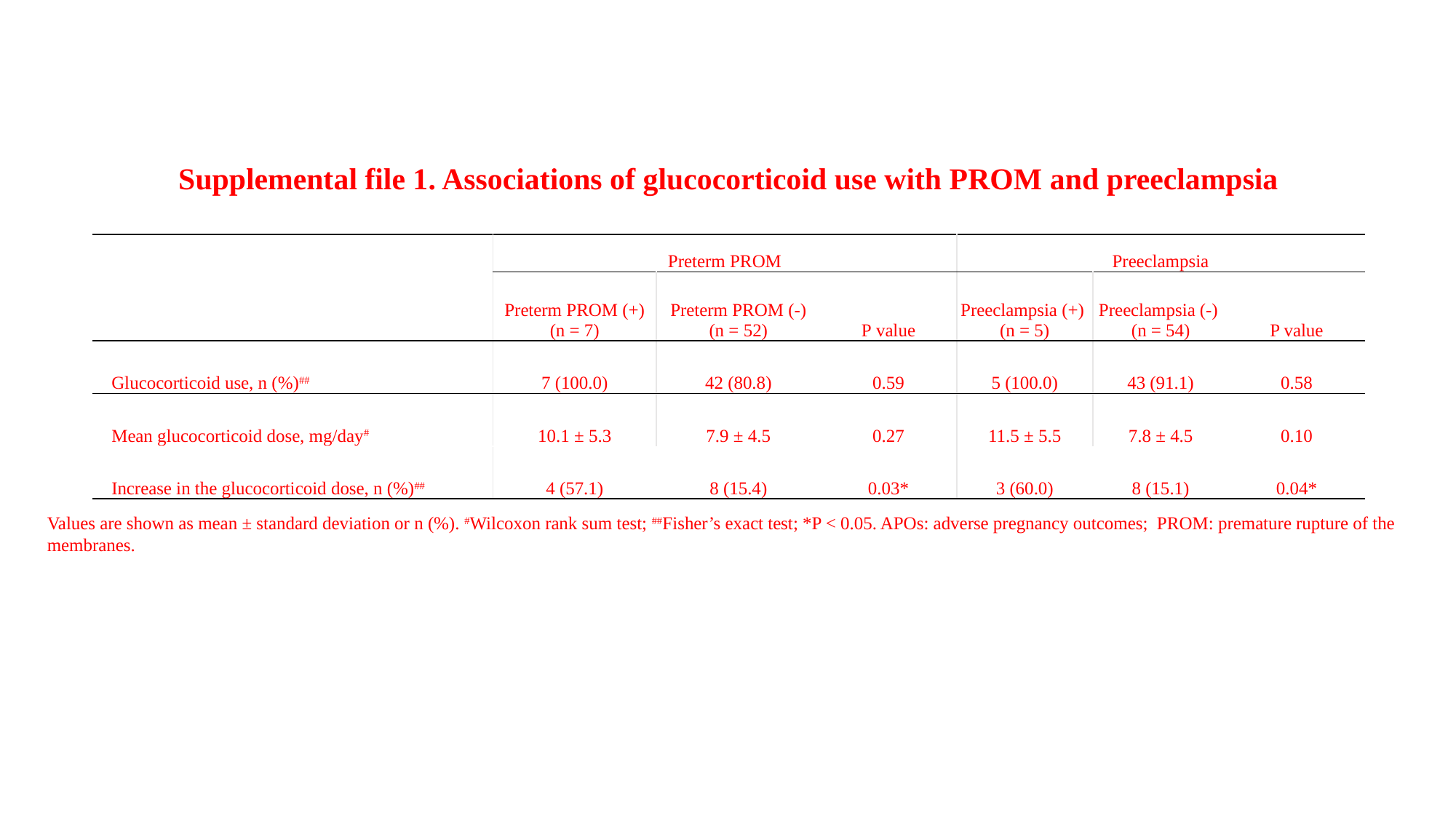

Supplemental file 1. Associations of glucocorticoid use with PROM and preeclampsia
| | Preterm PROM | | | Preeclampsia | | |
| --- | --- | --- | --- | --- | --- | --- |
| | Preterm PROM (+) (n = 7) | Preterm PROM (-) (n = 52) | P value | Preeclampsia (+) (n = 5) | Preeclampsia (-) (n = 54) | P value |
| Glucocorticoid use, n (%)## | 7 (100.0) | 42 (80.8) | 0.59 | 5 (100.0) | 43 (91.1) | 0.58 |
| Mean glucocorticoid dose, mg/day# | 10.1 ± 5.3 | 7.9 ± 4.5 | 0.27 | 11.5 ± 5.5 | 7.8 ± 4.5 | 0.10 |
| Increase in the glucocorticoid dose, n (%)## | 4 (57.1) | 8 (15.4) | 0.03\* | 3 (60.0) | 8 (15.1) | 0.04\* |
Values are shown as mean ± standard deviation or n (%). #Wilcoxon rank sum test; ##Fisher’s exact test; *P < 0.05. APOs: adverse pregnancy outcomes; PROM: premature rupture of the membranes.
